# Editorial: Greening the way: Emerging green technologies in process intensification

**DOI:** 10.3389/fchem.2024.1487667

**Published:** 2024-09-19

**Authors:** Georgios Psakis, Sholeem Griffin, Maria Dimopoulou, Athanasios Angelis-Dimakis, Jose Manuel Lorenzo

**Affiliations:** ^1^ Institute of Applied Sciences (IAS), The Malta College of Arts, Science & Technology (MCAST), Paola, Malta; ^2^ Metamaterials Unit, Faculty of Science, University of Malta (UM), Msida, Malta; ^3^ School of Health and Life Sciences (SHLS), Teesside University, Middlesbrough, United Kingdom; ^4^ School of Applied Sciences, Department of Physical and Life Sciences, University of Huddersfield, Huddersfield, United Kingdom; ^5^ Centro tecnolóxico da Carne (Ceteca), Ourense, Spain

**Keywords:** green technologies, green solvents, valorisation, sustainability, circular economy, ewaste, food waste, healthcare

## 1 Introduction

In 2022, 229.5 million metric tons of municipal solid waste (MSW; inclusive of electronic waste (e-waste), food waste, and healthcare waste) were generated in the European Union, amounting to 513 kg per person (European Commission, 2024). With the accumulation of MSW contributing to pollution, resource depletion, and greenhouse gas emissions (methane, carbon dioxide and nitrous oxide), further exacerbating climate change, effective waste management becomes essential for safeguarding environmental sustainability, public health and global resource security. The MSW management challenge has set in motion European plans (Circular Economy Action Plan (European Commission, 2020) and Green Deal (European Commission, 2021)) and global strategies (Sustainable Development Goal 12; Responsible Consumption and Production (United Nations Development Programme, 2021)), aiming at waste reduction, efficient/systematic recycling and resource recovery through green-valorisation (eco-friendly technologies with use of green-chemistry). Execution of these plans requires the adoption of innovative practices by multiple-stakeholders, planting seeds for the development of sustainable circular economies. Thus, application of Innovative tools and methods, and integration of time-, energy- and cost-effective technologies in process intensification, are now more critical than ever for attaining sustainability goals.

**FIGURE 1:**
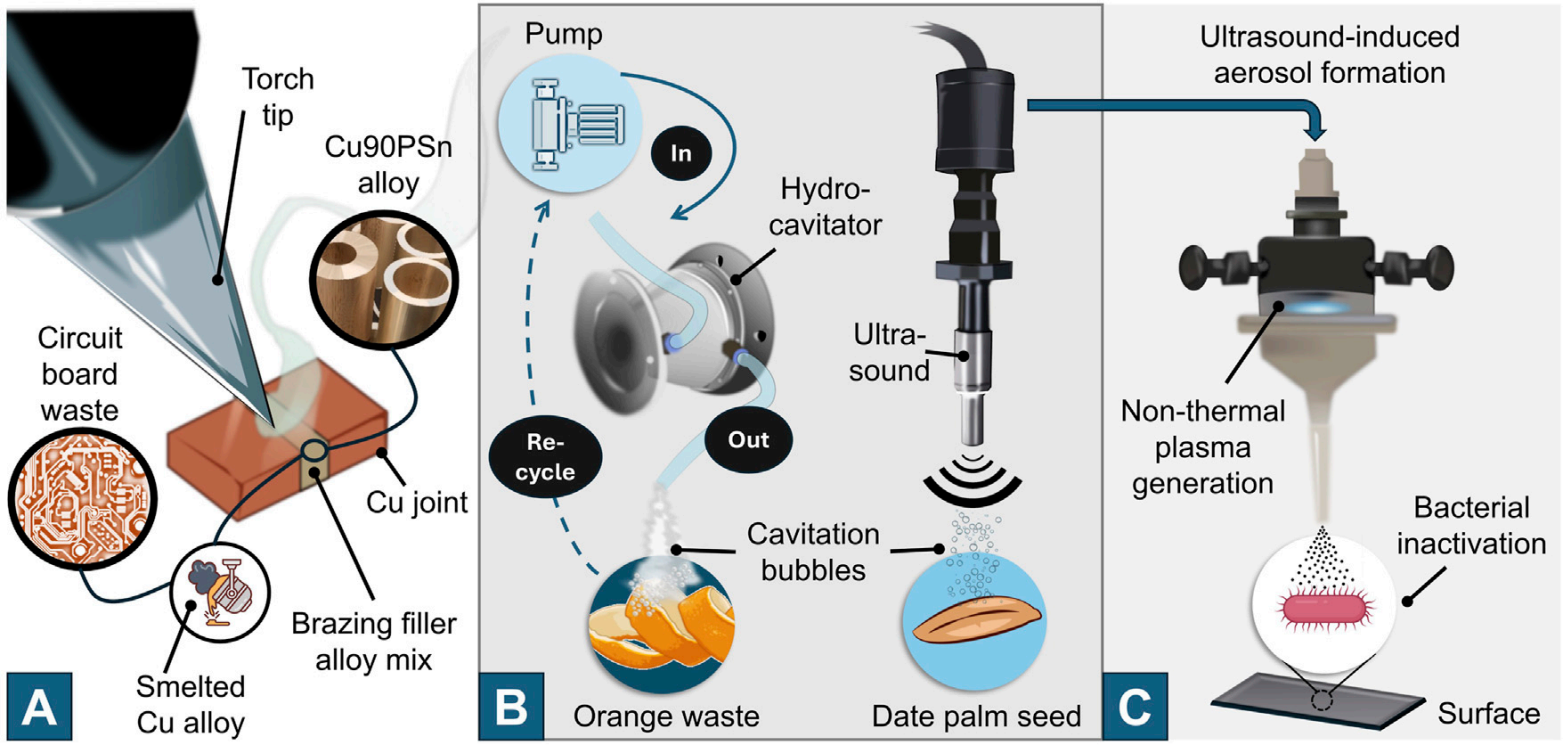
The green approaches to waste management covered in the Research Topic. Cu alloy smelting from circuit board waste and introduction to Cu90PSn brazing filler for Cu-Cu jointing (brazing rod not shown for clarity) **(A)**. Hydrodynamic cavitation-assisted extraction of bioactive compounds and valorisation of the processed peel (left) and ultrasound-assisted extraction of (poly)phenols from date palm seeds (right) **(B)**. Ultrasound-driven aerosol formation and non-thermal plasma aerosol activation for bacterial surface disinfection **(C)**.

The Research Topic “Greening the Way: Emerging Green Technologies in Process Intensification” highlights developments in 1) e-waste valorisation, 2) green-technologies for bioactive-compound extraction from food-waste, and 3) green technologies for microbial surface disinfection (Figure 1). Green technologies comprise practices that allow the harnessing of benefits (whether extraction of bioactive compounds from waste or the adoption of sustainable practices in processing and healthcare) by minimising and/or eliminating negative environmental impacts. The Research Topic also examines the time, energy, and cost efficiency of these approaches, and discusses factors influencing their effectiveness and adoption. Understanding advantages, limitations, and cause-and-effect relationships will aid the smooth integration of these technologies into existing processes.

## 2 Scope and significance

Green public procurement by local and national authorities plays a key role in advancing circular economies but relies heavily on research-based evidence to guide policy decisions. In alignment with the focus of this Research Topic, the presented scientific studies demonstrate how various technologies address current environmental challenges while underscoring their advantages in terms of time, cost, and energy efficiency compared to traditional methods. A central theme across the studies is the necessity of optimizing operational parameters for these technologies. In the context of electronic and food waste valorisation, the characteristics and composition of the input material, along with the targeted extraction outcome, determine the specific parameters requiring optimization. Similarly, electrode design in cold plasma systems influences the formation of reactive species for disinfection. Such optimizations are crucial for identifying conditions that maximize efficiency while minimizing time and energy consumption, offering essential insights for reactor selection and design when scaling up for industrial applications. Lastly, in food waste valorisation, workshops and living labs (Voglhuber-Slavinsky et al., 2023) reveal that stakeholders and policymakers often lack a full understanding of food-value chains, hindering networking and business model development due to difficulty in identifying key actors. The perspective by Psakis et al., at least for the citrus food chain, provides an example as to how different actors can come together to build such networks. It is such research insights that not only drive the development of valorisation and health service ecosystems, but they ultimately safeguard their viability, making economic circularity a reality (Alcalde-Calonge et al., 2022).

## 3 Scientific contributions

### 3.1 Effect of E-waste copper alloy additions on the microstructure and organization of Cu90PSn brazing joints

Bao et al. explore incorporating smelted copper alloys from e-waste (circuit boards) into Cu90PSn alloys for brazing copper-copper joints. The study assesses the wetting properties, tensile strength, and macro/microstructure of the joints after filler addition. They also identify the optimal content of e-waste-derived alloy in Cu90PS for achieving uniform brazing and maintaining welding performance.

### 3.2 Screening factors to affect ultrasound-assisted extraction of (poly)phenols from date palm seeds

Lucas-González et al. investigate ultrasound-assisted extraction of (poly)phenols from date palm seeds using green solvents, optimizing the process and comparing it to conventional methods. The study shows that extraction conditions depend on the specific (poly)phenols, with water favouring flavan-3-ols and ethanol quecertin. It also finds that the liquid-to-solid ratio limits extraction efficiency. The research emphasizes the time-effectiveness and eco-friendliness of ultrasound-assisted extraction over traditional methods.

### 3.3 A rapid prototyped atmospheric non-thermal plasma-activated aerosol device and anti-bacterial characterisation

de Oliveira Mallia et al., describe the production of a cold-plasma-activated ultrasound-generated aerosol (water dispersed in air), using a prototype set-up, whose components are fabricated by lithography 3D-printing for optimal device operation. The study demonstrates the bactericidal surface effects of the tested aerosols against *Escherichia coli*, *Pseudomonas aeruginosa*, *Staphylococcus aureus* and *Salmonella enterica*, and identifies nitrites, ozone, and low pH as the main determinants of the bactericidal propensity, particularly against Gram-negative bacteria, given the tested prototype.

### 3.4 Exploring hydrodynamic cavitation for citrus waste valorisation in Malta: from beverage enhancement to potato sprouting suppression and water remediation

Psakis et al., investigate the extraction of bioactive compounds from Citrus sinensis waste, using hydrodynamic cavitation, and provide a perspective for coupling the waste extraction with the valorisation of the (un)processed peel in potato sprouting prevention and wastewater remediation for agricultural irrigation in Malta. This perspective exemplifies one of many ways through which a circular-economy culture can emerge in countries lacking established juice-plant processing.

## 4 Conclusions and future directions

This issue advances extraction practices for natural compound recovery from fruit waste, and metal-alloys from e-waste, promoting sustainable food functionalisation, and material engineering, respectively. It also advances plasma technology in healthcare, offering disinfection solutions in a cost-effective and scalable manner, increasing the valuation of healthcare ecosystem services. Future actions for sustainability include:

• Development/maintenance of a centralized database with details on the selected green-technology, bench-scale/reactor setup/approach, purpose, optimal operation parameters, energy use, valorisation target, and environmental/health impact assessments.
• Funding boost for R&I/R&D to support industrial pilot projects and close the gap between lab research and commercial operations.
• Incentive provision for green start-ups.
• Life cycle assessments on both end- and side-stream valorisation-products, and economic feasibility studies to ensure environmental sustainability and commercial viability.
• AI/machine learning-algorithm use for predictive modelling, process optimization, and technology-integration into production streams.
• Promotion of evidence-informed policy making.
• Flexible technology legislation development based on valorization target and reactor type/approach.
